# TRENDS IN DESPAIR-RELATED CONDITIONS: A LOSS OF MENTAL HEALTH AND HOPE IN THE US 1995–2014

**DOI:** 10.1093/geroni/igad104.1960

**Published:** 2023-12-21

**Authors:** Anthony Bardo, Takashi Yamashita

**Affiliations:** University of Kentucky, Lexington, Kentucky, United States; University of Maryland, Baltimore County, Baltimore, Maryland, United States

## Abstract

The Deaths of Despair (DoD) narrative posits that increased prevalence of pain, psychological distress, and drug/alcohol misuse are linked to increased mortality rates among poor non-Hispanic whites in midlife. While data limitations prevent empirically linking these conditions with DoD mortalities, understanding whether period shifts in mental health and/or hope underly their increased prevalence is needed to inform program and policy directives. The present study used two waves (1995-1996 and 2011-2014) of the Midlife in the United States (MIDUS) study to examine trends in the association between these despair-related conditions with mental health and hope by age, race/ethnicity, and socioeconomic status (SES). Mental health was measured by the Complete Mental Health Continuum, and the negative end of this spectrum (i.e., languishing) was used as a proxy for “despair.” SES was based on an innovative index that is comparable across waves. Logit models with a despair x hope interaction term were used to identify demographic-group-specific period shifts. Results show that increased prevalence in these despair-related conditions is concentrated among low-SES groups, and standard decomposition techniques show that the largest drivers of an increased prevalence among these low-SES groups differs by race/ethnicity: among non-Hispanic whites it was a combination of despair and hopelessness, but hopelessness was the primary driver among racial/ethnic minorities. Findings are useful for targeting vulnerable groups in midlife and informing programs and policies to combat rising levels of pain, psychological distress, drug/alcohol misuse and related premature deaths.

